# Selective Toxicity of Secondary Metabolites from the Entomopathogenic Bacterium Photorhabdus luminescens *sonorensis* against Selected Plant Parasitic Nematodes of the Tylenchina Suborder

**DOI:** 10.1128/spectrum.02577-21

**Published:** 2022-02-09

**Authors:** Ayako Kusakabe, Chen Wang, Ya-ming Xu, István Molnár, S. Patricia Stock

**Affiliations:** a Graduate Interdisciplinary Program in Entomology and Insect Science, University of Arizona, Tucson, Arizona, USA; b School of Animal and Comparative Biomedical Sciences, University of Arizona, Tucson, Arizona, USA; c Southwest Center for Natural Products Research, University of Arizona, Tucson, Arizona, USA; d Biotechnology Research Institute, The Chinese Academy of Agricultural Sciences, Beijing, People's Republic of China; e VTT Technical Research Centre of Finland Ltd., Espoo, Finland; f College of Agriculture, California State University, Chico, California, USA; University of Minnesota

**Keywords:** *Photorhabdus*, secondary metabolites, *Meloidogyne*, *Tylenchulus*, nematicidal activity, synergy

## Abstract

Entomopathogenic *Photorhabdus* bacteria (Enterobacteriaceae: Gamma-proteobacteria), the natural symbionts of *Heterorhabditis* nematodes, are a rich source for the discovery of biologically active secondary metabolites (SMs). This study describes the isolation of three nematicidal SMs from *in vitro* culture supernatants of the Arizona-native Photorhabdus luminescens *sonorensis* strain Caborca by bioactivity-guided fractionation. Nuclear magnetic resonance spectroscopy and comparison to authentic synthetic standards identified these bioactive metabolites as *trans*-cinnamic acid (*t*-CA), (4*E*)-5-phenylpent-4-enoic acid (PPA), and indole. PPA and *t-*CA displayed potent, concentration-dependent nematicidal activities against the root-knot nematode (Meloidogyne incognita) and the citrus nematode (*Tylenchulus semipenetrans*), two economically and globally important plant parasitic nematodes (PPNs) that are ubiquitous in the United States. Southwest. Indole showed potent, concentration-dependent nematistatic activity by inducing the temporary rigid paralysis of the same targeted nematodes. While paralysis was persistent in the presence of indole, the nematodes recovered upon removal of the compound. All three SMs were found to be selective against the tested PPNs, exerting little effects on non-target species such as the bacteria-feeding nematode Caenorhabditis elegans or the entomopathogenic nematodes Steinernema carpocapsae, Heterorhabditis bacteriophora, and *Hymenocallis sonorensis*. Moreover, none of these SMs showed cytotoxicity against normal or neoplastic human cells. The combination of *t-*CA + PPA + indole had a synergistic nematicidal effect on both targeted PPNs. Two-component mixtures prepared from these SMs revealed complex, compound-, and nematode species-dependent interactions. These results justify further investigations into the chemical ecology of *Photorhabdus* SMs, and recommend *t-*CA, PPA and indole, alone or in combinations, as lead compounds for the development of selective and environmentally benign nematicides against the tested PPNs.

**IMPORTANCE** Two phenylpropanoid and one alkaloid secondary metabolites were isolated and identified from culture filtrates of *Photorhabdus l. sonorensis* strain Caborca. The three identified metabolites showed selective nematicidal and/or nematistatic activities against two important plant parasitic nematodes, the root-knot nematode (Meloidogyne incognita) and the citrus nematode (*Tylenchulus semipenetrans*). The mixture of all three metabolites had a synergistic nematicidal effect on both targeted nematodes, while other combinations showed compound- and nematode-dependent interactions.

## INTRODUCTION

In the United States, plants are subject to attack from over 50,000 different parasites and pathogens (1, 2). Among them are plant parasitic nematodes (PPNs), which cause billions of dollars in crop losses annually (3–5) and threaten food security (6, 7). In the southwestern U.S., the most significant PPN-generated losses to agriculture are caused by two species in the Tylenchina suborder. The root-knot nematode (Meloidogyne incognita) is an endoparasite of over 3,000 plant species (8–10) while the citrus nematode *Tylenchulus semipenetrans* is a semi-endoparasite of numerous citrus species worldwide (11, 12). In both species, the first-stage juveniles undergo one molt while still in the egg. The second stage juveniles (J2s) hatch from the egg and represent the infective stage. After penetrating the plant host through its roots, M. incognita J2s migrate through cortical tissues toward the vascular zone where they establish a permanent feeding site (i.e., giant cells), acquire a “sausage” shape and become sedentary (8, 9). Three additional molts occur prior to the adult stage. M. incognita is sexually dimorphic with globose shaped females that remain in the plant roots, while males are vermiform and leave the roots. Upon maturity, females lay eggs into a gelatinous mass (galls) that protect them against unfavorable environmental conditions, and the life cycle is repeated. In *T. semipenetrans*, only the anterior end of the young females penetrates the cortex of the root where these J2s begin feeding on three to six nurse cells. The intense feeding by the females causes their posterior end to enlarge and protrude outside the root. After fertilization, females lay eggs outside of the root in a gelatinous matrix and the life cycle is reinitiated (11).

At present, management of PPNs relies heavily on the use of synthetic nematicides (13–18). However, the deleterious effects of these chemicals on wildlife, the environment, and human health are well documented (19, 20). Moreover, the recent banning of several chemical nematicides and the phase-out of methyl bromide from the pest-control market severely limit the available chemical treatment options (21) for annual and perennial crops. These considerations highlight an urgent need to discover new, environmentally friendly methods for the cost-effective management of PPNs in agriculture.

A promising approach to develop such methods is to study microorganisms that antagonize PPNs by producing biologically active secondary metabolites (SMs; 17–20). Among these microorganisms, entomopathogenic *Photorhabdus* bacteria are considered a rich source of bioactive metabolites (22–26). *Photorhabdus* are the natural mutualistic symbionts of *Heterorhabditis* entomopathogenic nematodes. The third-stage infective juveniles (IJs) of these entomopathogenic nematodes carry the bacteria in their intestinal tract and vector them from one insect host to another (27). Once the *Heterorhabditis* IJs penetrate a new insect host, they migrate to the hemolymph and regurgitate *Photorhabdus* cells into the body cavity of the insect. There, the bacteria release potent toxins that kill the insect host within 24 h to 48 h. During this pathogenic phase, *Photorhabdus* also produce small molecule SMs that have broad-spectrum bioactivities to outcompete other microorganisms that may invade, or scavengers that would consume the insect cadaver (28).

Previous studies have shown that certain *Photorhabdus*-derived SMs exhibit antibacterial (29), antifungal (30, 31), insecticidal (32), and nematicidal activities (33–35). A body of evidence also suggests that the spectra of SM production differ widely among different *Photorhabdus* species and even strains (23, 36), and under different environmental and nutrient availability conditions (36). Furthermore, closely related strains may produce different SM congeners that may exhibit different biological activities (37, 38). Therefore, new *Photorhabdus* species and/or strains may yield novel SMs with diverse bioactivities (38), and may have the potential to be developed into bio-nematicides.

Over the past 2 decades, a wide variety of SMs have been isolated and structurally characterized from *Photorhabdus* strains. Nevertheless, only a few SMs including stilbenes, anthraquinones, indoles, peptides, and phenol derivatives have been tested for biological activity under laboratory conditions (30–35, 39, 40). These *Photorhabdus*-derived SMs were successfully isolated using two different *in vitro* approaches. Most of the early studies on *Photorhabdus*-derived SMs employed a biologically unbiased workflow to isolate prominent SMs under varied culture conditions (30, 40, 41), with the isolated SMs evaluated for selected biological activities in a *post hoc* analysis (30, 40). More recent studies adopted a chemically unbiased approach, and used bioassay-guided fractionation to isolate, purify, and structurally characterize bioactive SMs responsible for targeted bioactivities (31, 32). For some SMs with a known structure, total chemical synthesis may replace the isolation of metabolites from the microorganisms. Such “synthetic SMs” may facilitate bioactivity evaluations, especially when the productivity of the microbial cultures is insufficient. Moreover, synthetic SMs may also offer a more facile source for structure-activity relationship studies, and a low-cost alternative for the commercial production of active ingredients for pharmaceuticals and agrochemicals, including nematicides.

Until now, only a few studies explored the targeted isolation of *Photorhabdus* SMs with activities against PPNs (33–35). Thus, 3,5-dihydroxy-4-isopropylstilbene from *P. luminescens* MD displayed nematicidal activity against the targeted plant- or fungal-feeding nematodes such as *Bursaphelenchus* and *Aphelenchoides* spp. However, it was also toxic at similar concentrations (up to 100 μg/ml) to Caenorhabditis elegans, a non-target bacterivorous nematode. More importantly, 3,5-dihydroxy-4-isopropylstilbene showed no activity against the most economically significant PPNs such as the root-knot nematode, Meloidogyne incognita (33, 34). In contrast, indole, isolated from the same *P. luminescens* strain, provoked a transient paralysis on the tested nematode species, causing a nematicidal effect only at high concentrations. No results were reported for the effects of indole on C. elegans or entomopathogenic non-target nematodes (33, 34). These pioneering studies also did not formally determine the LC_50_ (lethal concentration causing 50% nematode death) or the EC_50_ values of the compounds (effective concentration causing 50% nematode reversible paralysis) against the infective juvenile stages of the tested nematodes.

Previously, our team assessed the bioactivities of crude extracts (complex mixtures of SMs) of two Arizona-native Photorhabdus luminescens strains (Caborca and CH35) to reveal selective nematicidal activities against the infective juveniles of the targeted PPN species, the root knot nematode *M. incognita* (23). These same extracts displayed low to no nematicidal activity against non-target soil nematode species, such as C. elegans and the entomopathogenic nematodes *Steinernema* spp. In addition, the LC-MS fingerprints of these crude extracts indicated the presence of at least nine unique compounds not previously detected in other *Photorhabdus* species. Based on these results, we hypothesized that these *Photorhabdus* strains may produce novel SMs with selective nematicidal activity against economically important PPNs, and that certain combinations of these SMs may even result in synergistic effects.

The present study describes the isolation and structure elucidation of three SMs with nematicidal activities from the Arizona-native *Photorhabdus* spp. Caborca strain. Specifically, the nematicidal and/or nematistatic activities of the individual compounds and SM combinations were assessed against two targeted PPN species, *M. incognita* and *T. semipenetrans*, both ubiquitous in the southwestern U.S. The effects of the metabolites were also evaluated against a panel of non-target nematodes, including the free-living stages of bacteria-feeding and entomopathogenic species. The influence of these compounds on human cells and the assessment of the synergistic, additive, and antagonistic effects of the SM mixtures *in vitro* are also reported.

## RESULTS

### Isolation and identification of nematicidal SMs.

**FIG 1:**
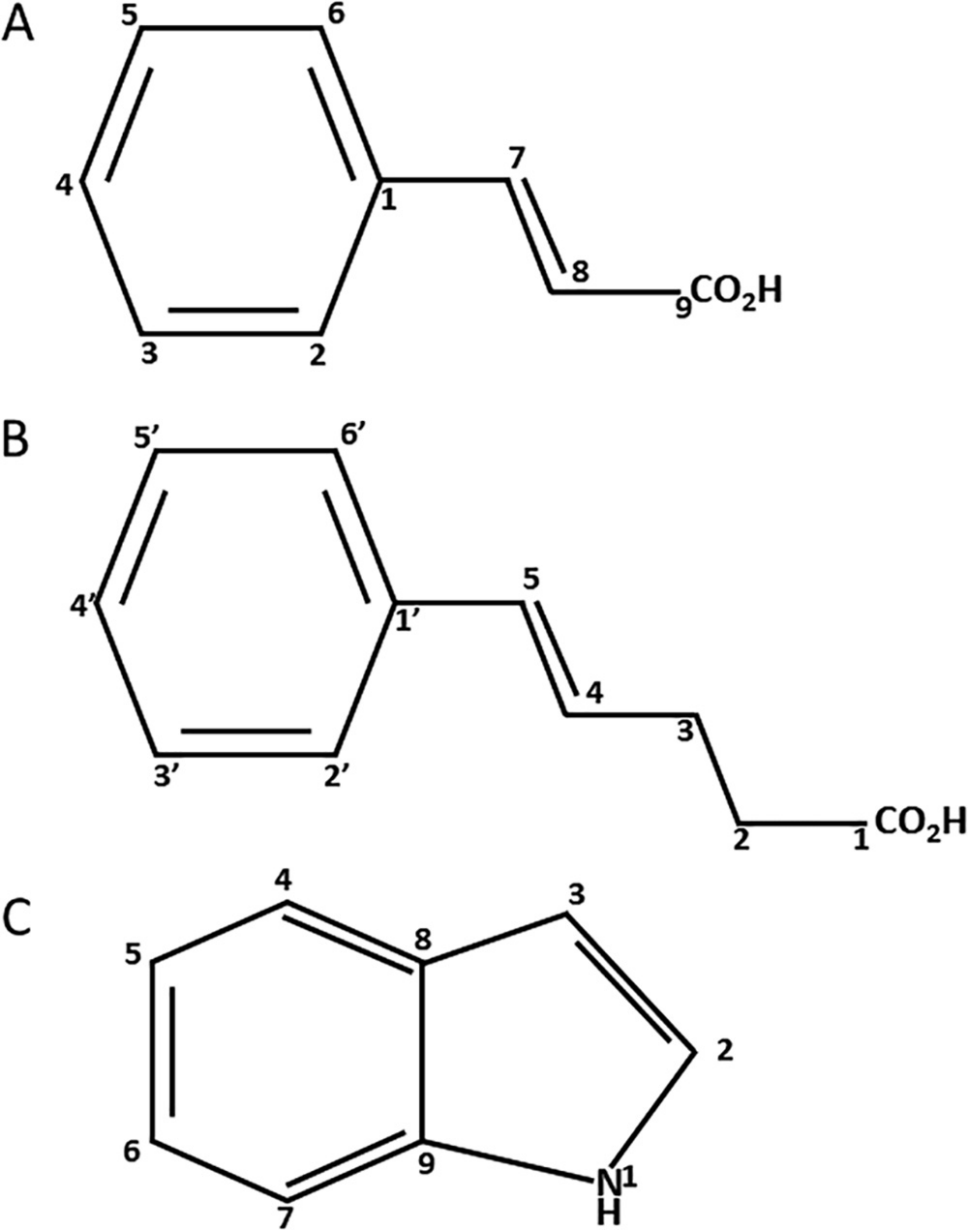
Nematicidal secondary metabolites from *P. l. sonorensis* Caborca. (A) *trans*-cinnamic acid (*t*-CA). (B) (4*E*)-5-phenylpent-4-enoic acid (PPA). (C) Indole.

Three metabolites with nematicidal activities were isolated from *in vitro* liquid cultures of *P. l. sonorensis* Caborca by bioactivity-guided fractionation, using the J2 of *M. incognita* as the indicator PPN species. The structures of pure, bioactive *Photorhabdus*-derived SMs were elucidated by NMR spectroscopy (Fig. 1; see also Tables S1 to S3 in the supplemental material, https://www.researchgate.net/publication/357662416_Selective_Toxicity_of_Secondary_Metabolites_from_the_Entomopathogenic_Bacterium_Photorhabdus_luminescens_sonorensis_against_Selected_Plant_Parasitic_Nematodes_of_the#fullTextFileContent) and by comparison to authentic synthetic standards. The metabolites were identified as: (i) *trans*-cinnamic acid (*t-*CA); (ii) (4*E*)-5-phenylpent-4-enoic acid (PPA); and (iii) indole.

### *trans-*cinnamic acid shows selective nematicidal activity against targeted PPNs.

**FIG 2:**
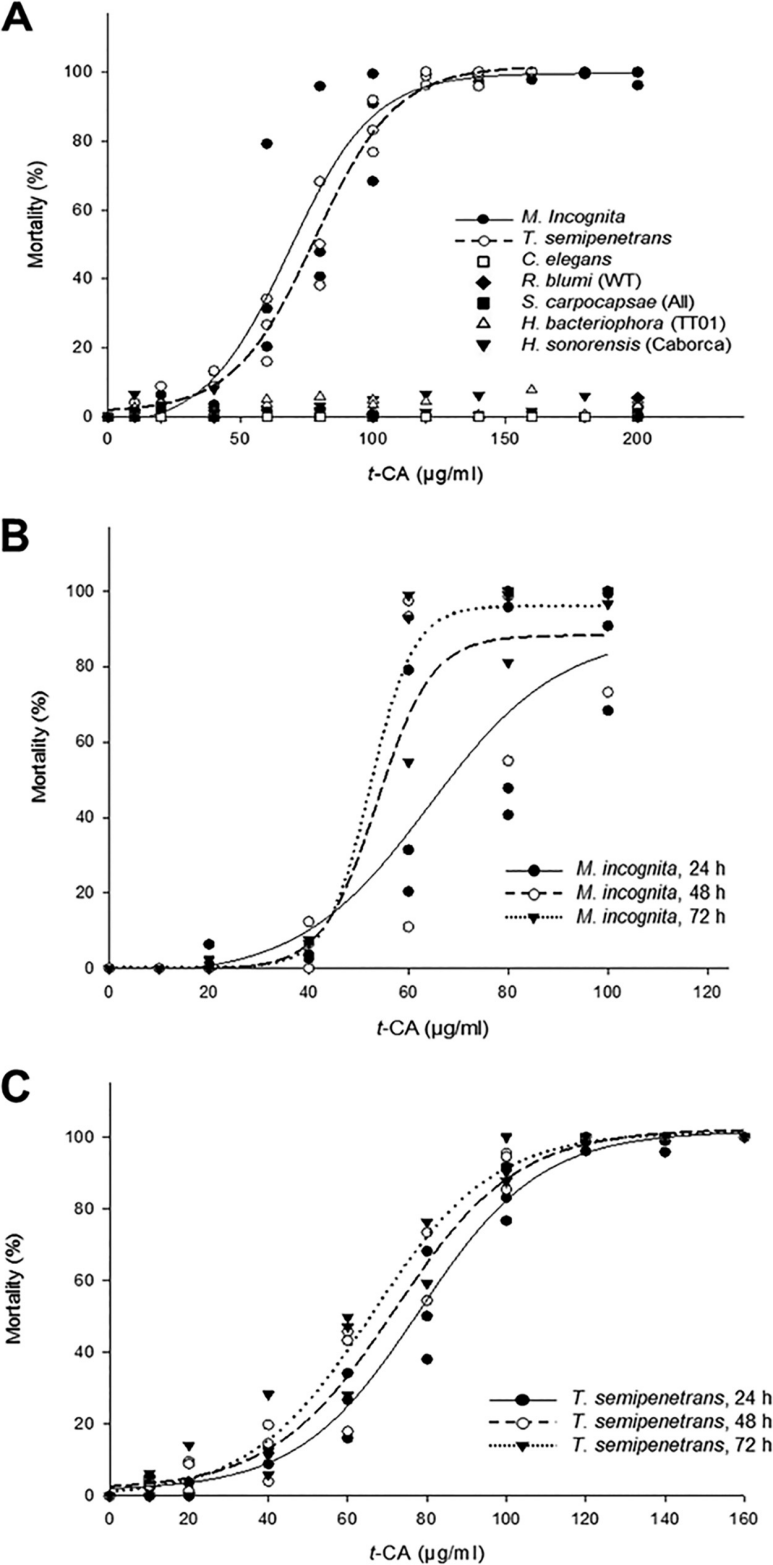
Nematicidal effects of *t*-CA. (A) Acute toxicity exerted by various concentrations of *t-*CA against the indicated nematodes after 24-h exposure. (B) Acute toxicity exerted by various concentrations of *t-*CA against *M. incognita* after 24-h, 48-h, or 72-h exposure. (C) Acute toxicity exerted by various concentrations of *t-*CA against *T. semipenetrans* after 24-h, 48-h, or 72-h exposure.

*t-*CA displayed a potent, concentration-dependent nematicidal activity against the targeted PPNs (Fig. 2A). At 24 h postexposure, the LC_50_ of *t*-CA was 67 μg/mL against M. incognita and 76 μg/ml against *T. semipenetrans* (Table 1). These LC_50_ values were not statistically different, as shown by the non-overlapping 95% confidence intervals of the LC_50_ values (Table 1) and corroborated by unpaired two-samples Wilcoxon tests (Table S4A).

**TABLE 1:** *In vitro* nematicidal activities of *t-*CA, PPA, and indole

Compound	Species	LC_50_^*a*^ [μg/mL]	95% CI^*c*^	Slope ± SE^*d*^
*t-*CA	*M. incognita*	67.3	(56.5, 77.2)	6.48 ± 0.29
	*T. semipetrans*	75.8	(67.4, 82.0)	8.29 ± 0.50
	C. elegans	308	(294, 322)	11.4 ± 0.40
	*S. carpocapsae*	>500^*e*^	—^*h*^	—
	*H. bacteriophora*	356	(317, 386)	9.82 ± 0.56
	*H. sonorensis*	421	(399, 440)	20.5 ± 1.06
	*R. blumi*	345	(333, 357)	16.6 ± 0.93
PPA	*M. incognita*	44.4	(38.8, 48.6)	7.19 ± 0.43
	*T. semipetrans*	66.3	(50.7, 73.4)	11.1 ± 0.79
	C. elegans	278	(244, 308)	11.6 ± 0.60
	*S. carpocapsae*	>400^*f*^	NA^*i*^	1.90 ± 0.94
	*H. bacteriophora*	>400^*f*^	NA	4.33 ± 0.59
	*H. sonorensis*	>400^*f*^	NA	0.46 ± 0.32
	*R. blumi*	346	(327, 363)	16.1 ± 0.93
Compound	Species	EC_50_^*b*^ [μg/mL]	95% CI^*c*^	Slope ± SE^*d*^
Indole	*M. incognita*	56.3	(52.9, 59.6)	9.48 ± 0.47
	*T. semipenetrans*	37.1	(28.7, 43.4)	5.37 ± 0.30
	C. elegans	159	(152, 168)	6.10 ± 0.22
	*S. carpocapsae*	>400^*g*^	NA	NA
	*H. bacteriophora*	>400^*g*^	NA	NA
	*H. sonorensis*	>400^*g*^	NA	NA
	*R. blumi*	37.0	(23.9, 48.3)	2.39 ± 0.10

a LC_50_, lethal concentration, causing 50% mortality to the nematode population after 24 h of *in vitro* exposure.

b EC_50_, effective concertation, causing 50% reversible paralysis to the nematode population after 24 h of *in vitro* exposure.

c 95% CI, 95% confidence interval for the LC_50_ or EC_50_.

d SE, standard error.

e 50% mortality was not reached after 24-h exposure at the highest concentration tested (500 μg/mL).

f 50% mortality was not reached after 24-h exposure at the highest concentration tested (400 μg/mL).

g 50% temporary paralysis was not reached after 24-h exposure at the highest concentration tested (400 μg/mL).

h —, not established.

i NA, not applicable.

Upon extended exposure to *t-*CA (24 h versus 48 h or 72 h), we observed a trend indicating that slightly lower *t-*CA concentrations may be required to kill 10%, 25%, 50%, and 90% of the two targeted nematode populations (Fig. 2B and C; Fig. S1), including a 34% reduction of the LC_90_ against M. incognita at 72 h versus 24 h (Table 2). However, these differences for the LC values at different time points did not reach statistical significance in most cases (Tables 2 and 3). A linear regression analysis and a Wilcoxon test further validated the observed non-significant effects of exposure times on *t-*CA potencies against the targeted PPNs (Fig. S1; Tables S5 and S6), although a linear decrease of the LC_10_ values was seen against M. incognita with a regression coefficient (b) of –.14 (*P = *0.02; Fig. S1B) as confirmed by the Wilcoxon test (Table S5). In addition, no differences were observed in the nematicidal potencies of *t*-CA between the targeted PPNs upon 48 h and 72 h of *in vitro* exposure (Tables S4B and S4C).

**TABLE 2:** *In vitro* activity of *Photorhabdus*-derived secondary metabolites against J2 juveniles of Meloidogyne incognita

Compound	Exposure	LC_10_^*a*^	LC_25_^*a*^	LC_50_^*a*^	LC_90_^*a*^	Slope ± SE^*d*^
		[μg/mL] (95% CI^*c*^)				
*t-*CA	24 h	42.7 (25.6, 52.2)	53.0 (38.0, 61.6)	67.3 (56.5, 77.2)	106.0 (89.6, 159.0)	6.48 ± 0.29
	48 h	37.3 (19.1, 47.2)	45.7 (29.0, 55.1)	57.3 (44.0, 68.2)	87.9 (73.0, 137.0)	6.89 ± 0.30
	72 h	39.5 (30.8, 45.1)	45.2 (37.7, 50.4)	52.5 (46.3, 58.0)	69.7 (62.6, 82.9)	10.4 ± 0.45
PPA	24 h	29.4 (22.1, 34.6)	35.8 (29.0, 40.5)	44.4 (38.8, 48.6)	66.9 (61.4, 75.5)	7.19 ± 0.43
	48 h	27.6 (19.0, 33.3)	33.6 (25.7, 39.0)	42.0 (35.4, 46.9)	63.9 (57.2, 75.9)	7.01 ± 0.39
	72 h	24.8 (16.0, 31.0)	30.7 (22.0, 36.6)	38.9 (31.2, 44.9)	61.0 (52.7, 76.5)	6.56 ± 0.33
		EC_10_^*b*^	EC_25_^*b*^	EC_50_^*b*^	EC_90_^*b*^	
Compound	Exposure	[μg/mL] (95% CI^*c*^)				
Indole	24 h	41.3 (36.8, 44.9)	47.8 (43.9, 51.1)	56.3 (52.9, 59.6)	77.0 (72.3, 83.2)	9.48 ± 0.47
	48 h	44.3 (36.8, 49.7)	51.7 (45.0, 56.6)	61.3 (55.8, 66.1)	84.7 (77.9, 95.3)	9.10 ± 0.43
	72 h	62.9 (31.4, 72.3)	68.9 (42.1, 76.9)	76.3 (57.7, 83.2)	92.5 (85.0, 119.0)	15.3 ± 1.27

a Lethal concentration (LC_10, 25, 50__, 90_), the concentration of the indicated compound necessary to cause 10%, 25%, 50%, or 90% mortality, respectively, in the *M. incognita* J2 juvenile populations after 24 h of *in vitro* exposure.

b Effective concentration (EC_10, 25, 50__, 90_), the concentration of the indicated compound required to cause temporary paralysis in 10%, 25%, 50%, or 90%, respectively, of the *M. incognita* J2 juvenile populations after 24 h of *in vitro* exposure.

c 95% CI, 95% confidence interval for the LC_50_ or EC_50_.

d SE, standard error.

**TABLE 3:** *In vitro* activity of *Photorhabdus*-derived secondary metabolites against J2 juveniles of *Tylenchulus semipenetrans*

Compound	Exposure	LC_10_^*a*^	LC_25_^*a*^	LC_50_^*a*^	LC_90_^*a*^	Slope ± SE^*d*^
		[μg/mL] (95% CI^*c*^)				
*t-*CA	24 h	53.1 (41.2, 61.2)	62.8 (52.2, 70.0)	75.8 (67.4, 82.0)	108.0 (100.0, 121.0)	8.29 ± 0.50
	48 h	51.5 (39.7, 59.1)	60.0 (49.6, 66.6)	71.0 (63.0, 76.8)	98.0 (90.7, 110.0)	9.17 ± 0.58
	72 h	46.2 (34.5, 54.0)	54.9 (44.4, 61.8)	66.5 (58.1, 72.5)	95.8 (88.2, 108.0)	8.10 ± 0.51
PPA	24 h	50.8 (27.0, 60.5)	57.6 (36.6, 66.0)	66.3 (50.7, 73.4)	86.4 (78.2, 109.0)	11.10 ± 0.79
	48 h	42.7 (31.2, 50.0)	50.1 (39.8, 56.6)	59.8 (51.7, 65.6)	83.7 (76.3, 96.6)	8.78 ± 0.52
	72 h	19.3 (3.06, 31.9)	28.0 (8.0, 42.7)	42.2 (20.9, 65.2)	92.4 (60.6, 312.0)	3.76 ± 0.12
		EC_10_^*b*^	EC_25_^*b*^	EC_50_^*b*^	EC_90_^*b*^	
Compound	Exposure	[μg/mL] (95% CI^*c*^)				
Indole	24 h	21.4 (12.8, 27.9)	27.8 (18.9, 34.2)	37.1 (28.7, 43.4)	64.2 (55.7, 77.8)	9.48 ± 0.47
	48 h	40.6 (8.18, 57.5)	51.2 (16.1, 67.0)	66.2 (33.5, 80.6)	108.0 (89.7, 173.0)	6.04 ± 0.44
	72 h	40.5 (13.1, 55.8)	51.9 (23.3, 66.0)	68.3 (43.5, 81.0)	115.0 (97.4, 175.0)	5.65 ± 0.38

a Lethal concentration (LC_10, 25, 50__, 90_), the concentration of the indicated compound necessary to cause 10%, 25%, 50%, or 90% mortality, respectively, in the *T. semipenetrans* J2 juvenile populations after 24 h of *in vitro* exposure.

b Effective concentration (EC_10, 25, 50__, 90_), the concentration of the indicated compound required to cause temporary paralysis in 10%, 25%, 50%, or 90%, respectively, of the *T. semipenetrans* J2 juvenile populations after 24 h of *in vitro* exposure.

c 95% CI, 95% confidence interval for the LC_50_ or EC_50_.

d SE, standard error.

*t-*CA exerted weak nematicidal activity against the selected non-target nematodes after 24 h of exposure at concentrations above 200 μg/mL (Fig. 2A and Table 1). Mortality with one of the non-target entomopathogenic nematodes, *S. carpocapsae*, did not reach 50% even at the highest TCA concentration tested (500 μg/mL), so an LC_50_ could not even be established (Table 1). The LC_50_ values against the other non-target entomopathogenic nematodes (*H. bacteriophora* and *H. sonorensis*) and the bacteria-feeding nematodes (C. elegans and *Rhabditis blumi*) ranged from 308 to 421 μg/mL, with the highest LC_50_ value measured for *H. sonorensis*, the natural host of the *t-*CA producer *P. l. sonorensis* Caborca (Table 1). Thus, the LC_50_ values of *t-*CA against the targeted PPNs at 24 h of exposure were significantly lower than those observed against the non-target nematodes (Table S4A), with this compound displaying a selectivity index of ≥ 5-6 (LC_50_ for the non-target nematodes over those for the targeted PPNs). Notably, the 48-h and 72-h LC_50_ values of *t*-CA against the targeted PPNs were also significantly lower than the 24-h LC_50_ of *t*-CA against the selected non-targeted nematodes (Tables S4B and S4C).

### (4*E*)-5-phenylpent-4-enoic acid (PPA) shows selective nematicidal activity against targeted PPNs.

**FIG 3:**
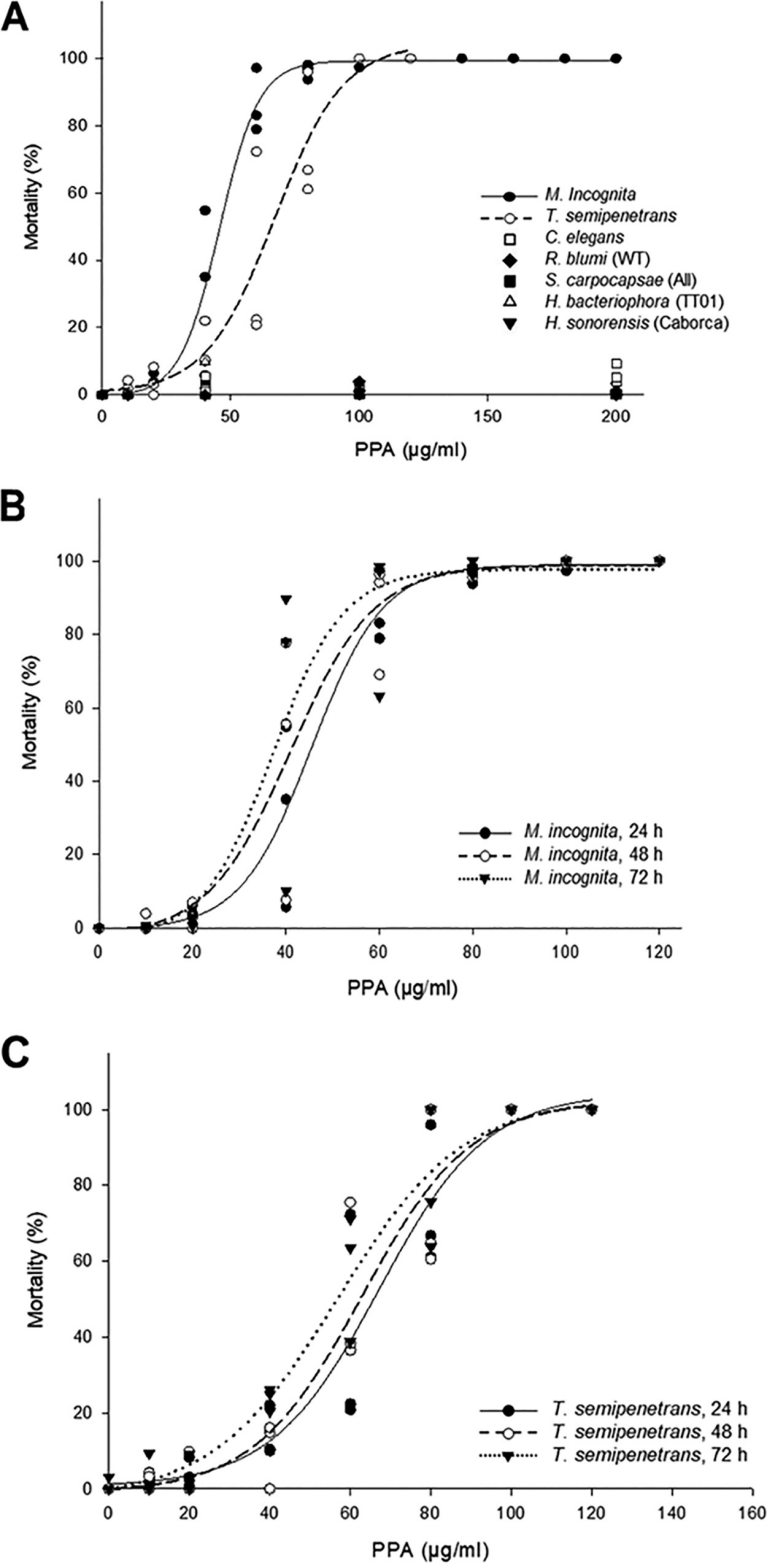
Nematicidal effects of PPA. (A) Acute toxicity exerted by various concentrations of PPA against the indicated nematodes after 24-h exposure. (B) Acute toxicity exerted by various concentrations of PPA against *M. incognita* after 24-h, 48-h, or 72-h exposure. (C) Acute toxicity exerted by various concentrations of PPA against *T. semipenetrans* after 24-h, 48-h, or 72-h exposure.

PPA also exhibited potent, concentration-dependent nematicidal activity against the targeted PPNs (Fig. 3A). The LC_50_ value of PPA for M. incognita (44 μg/mL) was significantly lower (a 33% reduction) than that observed for *T. semipenetrans* (66 μg/mL) at 24 h postexposure (Table 1; Table S4A). When considering extended exposure to this metabolite, a trend was perceivable in most cases for lower concentrations of PPA that were required to kill 10%, 25%, 50%, and 90% of the two target nematode populations after 48 h and 72 h of exposure (Fig. 3B and C; Fig. S2). However, these trends did not reach statistical significance against either of the two targeted PPNs (Tables 2 and 3). A linear regression analysis and a Wilcoxon test led to the same conclusion when considering *M. incognita* as the target (Fig. S2A and Table S5), while the LC_10_ and the LC_25_ values against *T. semipenetrans* showed a modest linear decrease (LC_10_: b = −0.40, *P = *0.03; and LC_25_: b = −0.44, *P = *0.04; Fig. S2B). Likewise, significant decreases in the 48-h and 72-h LC_10_ values were observed when using the Wilcoxon test (Table S6). After being exposed to PPA from 48 h to 72 h, no significant differences in the nematicidal potencies of PPA were detected between the two targeted PPNs (Tables S4B and S4C).

PPA showed weak nematicidal activity against the non-target, bacteria-feeding nematode species at 24 h of exposure at concentrations above 200 μg/mL (Fig. 3A and Table 1). Thus, the LC_50_ value of PPA against C. elegans was found to be 278 μg/mL, while that for *R. blumi* was significantly higher (346 μg/mL). In contrast, LC_50_ values could not even be established against the selected entomopathogenic nematodes, because mortality did not reach 50% even at the highest PPA concentrations tested (400 μg/mL; Table 1). Thus, the 24-h LC_50_ values of PPA against the targeted PPNs were significantly lower than those observed against the non-target nematodes (Table S4A), with this compound displaying a selectivity index of ≥ 6 to 8. Also, the 48-h and 72-h LC_50_ values of PPA against the targeted PPNs were significantly lower than the 24-h LC_50_ against the selected non-targeted nematodes (Tables S4B and S4C).

### Indole shows selective nematistatic activity against targeted PPNs.

**FIG 4:**
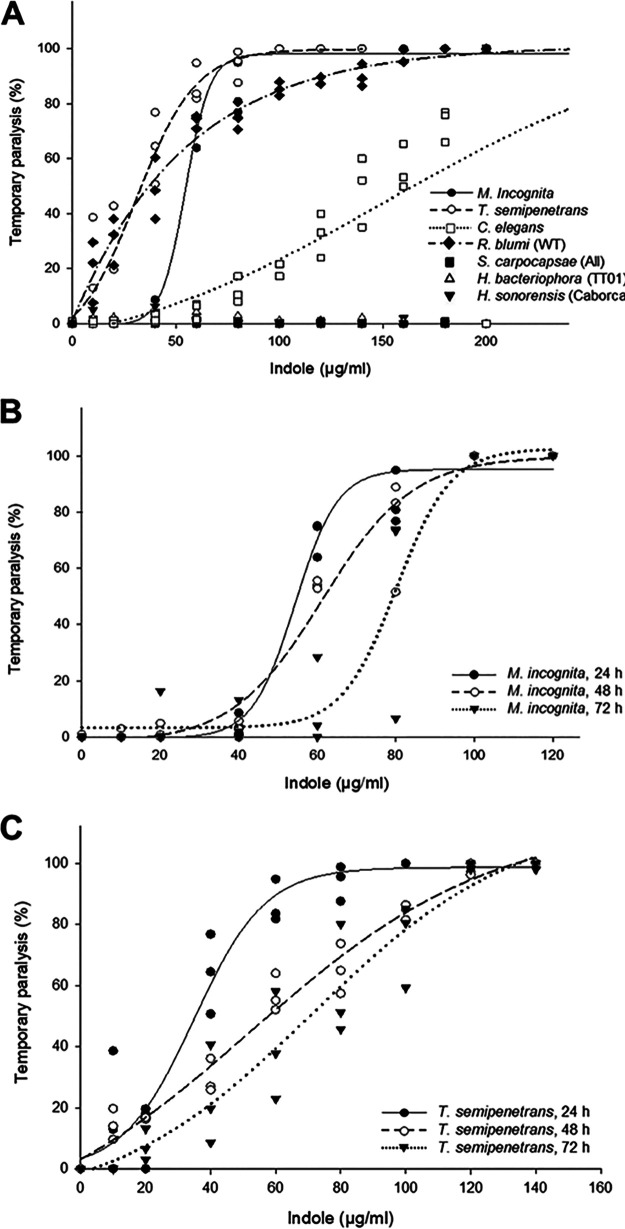
Nematistatic effects of indole. (A) Temporary paralysis exerted by various concentrations of indole against the indicated nematodes after 24 h exposure. (B) Temporary paralysis exerted by various concentrations of indole against *M. incognita* after 24-h, 48-h, or 72-h exposure. (C) Temporary paralysis exerted by various concentrations of indole against *T. semipenetrans* after 24-h, 48-h, or 72-h exposure.

Indole exhibited potent, concentration-dependent nematistatic activity against the targeted PPNs at 24 h postexposure (Fig. 4A), manifesting in temporary rigid paralysis of the nematodes. The EC_50_ of indole for *T. semipenetrans* (37 μg/mL) was significantly lower (34% reduction) than that observed for M. incognita (56 μg/mL) after 24 h of exposure (Table 1; Table S4A).

When considering extended indole exposure (up to 72 h), a trend was observed for increasing concentrations of indole to be necessary to sustain paralysis with increasing time. Judged by the non-overlapping 95% confidence intervals of the LC_50_ values, these trends were not statistically significant for M. incognita (Table 2), while for *T. semipenetrans*, both the EC_50_ and the EC_90_ were significantly higher (84% and 79% increase, respectively) at 72 h compared with that observed at 24 h (Table 3). However, a linear regression analysis and a Wilcoxon test suggest that these increasing trends are in fact significant for both of the targeted PPNs (Fig. S3 and Tables S5 and S6), with the exception of the EC_90_ against M. incognita (b = 0.16; *P* = 0.13; Fig. S3A). Indeed, the use of non-overlapping 95% confidence intervals to determine the significance of trends is deemed potentially too conservative in some cases (42). After 48 h and 72 h of the indole exposure, the nematistatic potency of this compound did not vary between the targeted PPNs (Tables S4B and S4C).

The targeted M. incognita J2s were able to recover from indole-induced temporary paralysis. When the paralyzed nematodes were rinsed with water after 24 h of indole exposure, between 93% (60 μg/mL of indole) to 96% (80 μg/mL of indole) of M. incognita regained baseline mobility after 24 h of recovery (χ^2^ = 4.80; *df* = 2; *P* = 0.09; Table 4). Extended exposure (72 h) to the same concentrations of indole yielded similar recovery rates (χ^2^ = 5.00; *df* = 2; *P* = 0.08).

**TABLE 4:** Recovery of Meloidogyne incognita J2 exposed to indole

Treatment	Recovery after 24 h in water [%]	
	24-h exposure to treatment	72-h exposure to treatment
Control (DMSO)	98.0	98.5
60 μg/mL indole	95.9	95.5
80 μg/mL indole	94.9	92.8
		
χ^2^	4.80	5.00
df^*a*^	2	2
*P*-value	0.09	0.08

a df, degree of freedom.

Indole also induced temporary paralysis on the selected non-target, bacteria-feeding nematodes (C. elegans and *R. blumi*; Fig. 4A). The 24-h EC_50_ of 37 μg/mL against *R. blumi* was significantly lower (a 77% reduction) than that observed against C. elegans (159 μg/mL) (Table 1; Table S4A). In contrast, the selected non-target entomopathogenic nematode species were resistant to the nematistatic effects of indole in concentrations up to 400 μg/mL.

The 24-h EC_50_ values of indole against the targeted PPNs were significantly lower than those observed against the non-target nematodes (Table S4A). There was also a significant reduction in the 24-h EC_50_ values against the targeted PPNs when compared to those against the entomopathogenic nematodes alone (Table S4A), but the difference did not reach statistical significance against the bacteria-feeding nematodes (Table S4A). Indole displayed a selectivity index of 5–8 when compared to the selected non-target nematode species (7-11 to the entomopathogenic nematodes alone and 2–3 to the bacteria-feeding nematodes alone). These observations indicate that the nematistatic activity of indole is highly selective when contrasting the targeted PPNs with the selected entomopathogenic nematodes, but this selectivity is much more limited with respect to the bacteriovores used in this study. Noticeably, the 48-h and 72-h EC_50_ values of indole against the targeted PPNs were still significantly lower than the 24-h EC_50_ against the selected non-target nematodes or against the selected entomopathogenic nematodes alone (Tables S4B and S4C).

The effects of indole could also be escalated to a nematicidal outcome with much higher concentrations of the compound. Thus, indole displayed an LC_50_ of 307 μg/ml against *M. incognita* at 24 h exposure, while the LC_50_ against *T. semipenetrans* was significantly higher than that observed against *M. incognita* (388 μg/ml; Tables 5 and 6). Indole also showed nematicidal activity against C. elegans and *R. blumi* at concentrations of ≥ 300 μg/ml (data not shown).

**TABLE 5:** Interaction effects among *Photorhabdus*-derived secondary metabolites in Meloidogyne incognita

Compound or mixture	LC_50_^*a*^ [μg/mL]	95%cl^*b*^ [μg/mL]	Slope (± SE^*c*^)	Additive index^*d*^ (CI^*b*^)	Combination effect
Individual compounds					
*t-*CA	67.3	(56.5, 77.2)	6.48 (± 0.29)	NA^*f*^	
PPA	44.4	(38.8, 48.6)	7.19 (± 0.43)	NA	
Indole	307.0	(277.0, 338.0)	23.0 (± 0.69)	NA	
Compound mixtures					
*t-*CA + indole	72.1^*e*^	63.0, 79.7^*e*^	5.31 (± 0.12)	–0.31 (–0.34 to −0.27)	Antagonistic interaction
*t-*CA + PPA	22.9^*e*^	16.7, 28.7^*e*^	3.18 (± 0.12)	0.17 (0.04 to 0.38)	Synergistic interaction
PPA + indole	40.0^*e*^	22.8, 45.6^*e*^	8.97 (± 0.87)	–0.03 (–0.07 to 0.49)	Additive interaction
*t-*CA + PPA + indole	19.9^*e*^	14.2, 26.5^*e*^	6.45 (± 0.31)	0.24 (0.03 to 0.49)	Synergistic interaction

a Lethal concentration, the concentration causing 50% mortality in the nematode population after 24 h of *in vitro* exposure.

b 95% confidence interval for the LC_50_.

c SE, standard error.

d Additive index values with confidence intervals in the negative range indicate antagonistic interactions; those in the positive range show synergetic interactions; and those with confidence intervals overlapping 0 signify additive interactions among the compounds in the mixtures.

e Equal concentrations (w/v) of two or three SMs were used in a 1:1 or 1:1:1 mixture. For example, a *t-*CA + PPA mixture of 72.1 μg/mL indicates a solution that contained 72.1 μg/mL *t-*CA and 72.1 μg/mL PPA.

f NA, not applicable.

**TABLE 6:** Interaction effects among *Photorhabdus*-derived secondary metabolites in *Tylenchulus semipenetrans*

Compound or mixture	LC_50_^*a*^ [μg/mL]	95%cl^*b*^ [μg/mL]	Slope (± SE^*c*^)	Additive index^*d*^ (CI^*b*^)	Combination effect
Individual compounds					
*t-*CA	75.8	(67.4, 82.0)	8.29 (± 0.50)	NA^*f*^	
PPA	66.3	(50.8, 73.4)	11.1 (± 0.79)	NA	
Indole	388.0	(378.0, 399.0)	20.0 (± 1.96)	NA	
Compound mixtures					
*t-*CA + indole	91.7^*e*^	(60.3, 103.0)^*e*^	9.17 (± 0.70)	–0.45 (–0.51 to −0.05)	Antagonistic interaction
*t-*CA + PPA	40.6^*e*^	(38.3, 42.6)^*e*^	14.3 (± 1.09)	–0.15 (–0.32 to −0.10)	Antagonistic interaction
PPA + indole	66.9^*e*^	(60.3, 71.7)^*e*^	8.94 (± 0.50)	–0.18 (–0.35 to −0.16)	Antagonistic interaction
*t-*CA + PPA + indole	27.3^*e*^	(12.4, 34.9)^*e*^	5.66 (± 0.43)	0.19 (0.01 to 1.17)	Synergistic interaction

a Lethal concentration, the concentration causing 50% mortality in the nematode population after 24 h of *in vitro* exposure.

b 95% confidence interval for the LC_50_.

c SE, standard error.

d Additive index values with confidence intervals in the negative range indicate antagonistic interactions; those in the positive range show synergetic interactions; and those with confidence intervals overlapping 0 signify additive interactions among the compounds in the mixtures.

e Equal concentrations (w/v) of two or three SMs were used in a 1:1 or 1:1:1 mixture. For example, a *t-*CA + indole mixture of 91.7 μg/mL indicates a solution that contained 91.7 μg/mL *t-*CA and 91.7 μg/mL indole.

f NA, not applicable.

### Mixtures of *t-*CA, PPA and indole exhibit varied interactions against target PPNs.

Different combinations of the three purified *P. l. sonorensis* SMs had different effects on the two targeted PPN species, ranging from antagonistic to synergistic (Tables 5 and 6). In *M. incognita*, the LC_50_ of the *t-*CA + PPA combination was 22.9 μg/ml, with no statistically significant change upon the further addition of indole (LC_50_ of 19.9 μg/mL for *t-*CA + PPA + indole) or upon the replacement of *t-*CA with indole (LC_50_ of 40.0 μg/mL for PPA + indole; Table 5). The potencies of the TCA + PPA two-compound mixture and the *t-*CA + PPA + indole three-compound combination were significantly higher than those of indole or *t-*CA used as individual SMs (Table 5; orthogonal contrast comparisons shown in Table S7). These two sets of compound mixtures also indicated synergistic effects against M. incognita, with an additive index (AI) of 0.17 (*t-*CA + PPA) and 0.24 (*t-*CA + PPA + indole) as calculated with the equation used for mixture toxicity evaluations (43). The PPA + indole mixture showed an additive effect (AI,– 0.03), while the *t-*CA + indole combination was antagonistic (AI, −0.31; Table 5) against M. incognita.

In *T. semipenetrans*, the LC_50_ of the *t-*CA + PPA + indole three-part combination (27.3 μg/mL) and that of the *t-*CA + PPA two-part combination (40.6 μg/mL) were significantly lower than those observed for each of the constituent metabolites (Table 6). The three-compound mixture was also significantly more potent than the *t-*CA + indole or the PPA + indole two-part combinations (Table 6; Table S8). Based on the equation used for the mixture toxicity evaluation (43), this three-component SM mixture had a synergistic effect against *T. semipenetrans*, with an AI of 0.19. The *t-*CA + PPA and the PPA + indole two-component mixtures exhibited weakly antagonistic effects (AI, −0.15), while the combination of *t-*CA + indole was strongly antagonistic (AI, −0.45; Table 6).

### *t-*CA, PPA, and indole display no *in vitro* toxicity against human cells.

None of the three isolated SMs from *P. l. sonorensis* Caborca displayed *in vitro* cytotoxicity against non-neoplastic human cells (HFF, foreskin cells), and against three human cancer cell lines (NCI-H460, non-small cell lung cancer; SF-268, central nervous system glioma; and MCF-7, breast cancer), even at the highest concentrations tested (200 μM = 30 μg/mL of *t*-CA, 35 μg/mL of PPA, 23 μg/mL of indole; data not shown).

## DISCUSSION

In this study, three secondary metabolites (SMs) with nematicidal activities were isolated from *in vitro* cultures of *P. luminescens sonorensis* strain Caborca, a bacterial symbiont of the Arizona-native entomopathogenic nematode *Heterorhabditis sonorensis* Caborca. The identities of the isolated metabolites as *trans*-cinnamic acid (*t-*CA), (4*E*)-5-phenylpent-4-enoic acid (PPA), and indole, respectively, were confirmed by ^1H-NMR^ and ^13^C-NMR spectroscopy (Tables S1 to S3) and comparison to authentic synthetic standards. While these SMs have previously been detected in cultures of other *P. luminescens* strains (31, 33–35, 41), detailed investigations of the concentration-dependent nematicidal and/or nematistatic activities of these compounds and their mixtures against plant parasitic nematodes (PPNs) have not been reported.

We found that both *t-*CA and PPA display potent nematicidal activities against the J2 of two economically important PPN species, M. incognita and *T. semipenetrans*, with the 24-h LC_50_ values in the 44 to 76 μg/mL range (Tables 1 to 3) and with the 48-h and 72-h LC_50_ values in the 39–66 μg/mL range (Tables 1 to 3). The LC_50_ values against the selected PPNs were significantly lower than those observed against the selected non-target nematode species (Tables 1 to 3; Tables S4A to S4C). While the nematicidal potency of *t-*CA did not differ significantly between the two targeted PPNs across observation times, PPA was 33% more potent against M. incognita than against *T. semipenetrans* at 24 h of *in vitro* exposure. However, when the incubation period was prolonged from 24 h to 48 or 72 h, the differences in the nematicidal potency of PPA against the target nematodes were no longer significant. Our investigation of the time dependence of the nematicidal effects of *t-*CA and PPA show that most mortality occurs within the first 24 h of exposure of *T. semipenetrans* and M. incognita (Tables 2 and 3; Fig. S1 and S2; Tables S5 and S6). This may indicate that both SMs are concentration-dependent nematicides that cause sudden-onset acute toxicity, although the mechanism of action of these SMs needs to be determined in further studies. Importantly, neither *t-*CA nor PPA had any effect, at concentrations corresponding to their LC_50_ values in M. incognita or *T. semipenetrans*, against a panel of non-target nematodes including the bacteria-feeding species C. elegans and *R. blumi*, and the entomopathogenic nematodes *H. bacteriophora*, *H. sonorensis*, and *S. carpocapsae* (Fig. 2A and 3A). *t-*CA and PPA elicited mortality only at high concentrations against these non-target nematodes (LC_50_ values in the range of 278–421 μg/mL; Table 1). For PPA, LC_50_ values could not be established for the entomopathogenic nematode species even at concentrations close to the water solubility limit of the compound (400 μg/mL). Moreover, neither *t-*CA nor PPA displayed *in vitro* cytotoxicity against human cells.

Isolation and structure elucidation of PPA as a constituent of the fermentation extracts of a Korean-native *Photorhabdus luminescence* [*sic*] strain was reported earlier (41). However, to the best of our knowledge no previous studies have investigated the activity of PPA against economically important PPNs. In contrast, *t-*CA has been shown to display nematistatic activity against the J2s of M. incognita and the potato cyst nematode *Globodera pallida* (44). Our results extend and refine these previous observations on *t-*CA and PPA by focusing on nematode mortality as the endpoint, and also reveal their selective nematicidal activity against both M. incognita and *T. semipenetrans* compared with a panel of non-target nematodes. Taken together, the potent and selective nematicidal activity of *t-*CA and PPA raises the possibility that these SMs may be suitable lead compounds to develop selective nematicides against these PPNs that cause multibillion dollar crop losses annually (3–5), and threaten food security worldwide (6, 7).

With respect to indole, previous studies have noted nematicidal effects at high concentrations, and paralysis at lower concentrations against tested PPNs (34, 45). In addition, Hu et al. (34) reported that the selected PPNs, *Bursaphelenchus xylophilus* and M. incognita, were more susceptible to indole than the non-target entomopathogenic nematodes such as *Heterorhabditis* spp. Here, we show that indole provokes nematistatic effects on the targeted PPNs *T. semipenetrans* and M. incognita, with EC_50_ of 37 or 56 μg/mL, respectively (Table 1). While *R. blumi* was similarly sensitive (EC_50_ of 37 μg/mL) to the nematistatic effects of indole, C. elegans, the other non-target bacteria-feeding nematode in our panel was much more resistant (EC_50_ of 159 μg/mL; Table 1; Table S4). With concentration escalation, nematicidal effects could eventually be elicited against the targeted PPNs and the selected bacterivores, but only at very high concentrations (LC_50_ of ≥ 300 μg/mL). Notably, indole displayed no significant nematistatic or nematicidal activities against any of the tested entomopathogenic nematodes even at concentrations near its limit of water solubility (Table 1), nor did it exert *in vitro* toxicity against human cells.

The onset of paralysis upon indole exposure was rapid, and this nematistatic effect was persistent against the studied PPNs up to 72 h, with only a very modest reduction in potency observed upon prolonged incubation with the compound (Tables 2 and 3; Fig. S3; Tables S5 and S6), attributable to degradation (45). Even at extended exposures, significantly lower EC_50_ values were found against the targeted nematodes, compared to the 24-h EC_50_ against the selected non-targeted nematodes. The increase of the EC_50_ values against the targeted PPNs upon increased exposure periods, indicates that indole is predominantly a concentration-dependent nematistatic agent, where paralysis can only be sustained with increased indole concentrations. The temporary nature of the paralysis caused by indole was also noted for *B. xylophilus* and M. incognita (34). Correspondingly, our experiments showed that M. incognita recovered from indole-induced temporary paralysis when the metabolite was removed, with over 90% of M. incognita regaining baseline mobility during a recovery period of 24 h, regardless of the length of prior exposure (24 h to 72 h) to the metabolite (Table 4).

The three nematicidal and/or nematistatic SMs investigated in this study are all readily produced by *P. l. sonorensis* Caborca under identical *in vitro* culture conditions. This raised the intriguing possibility that these SMs may also display synergistic interactions against the targeted species when they are used in combination. Here, we report the effects of two- or three-component mixtures of pure SMs on the targeted, economically important PPNs. Our findings reveal complex interactions that lead to different treatment outcomes based on the nematode species and/or the specific combinations of the metabolites used. Most importantly from a crop protection perspective, the three-component mixture (*t-*CA + PPA + indole) displayed a synergistic effect on both PPN species tested (Tables 5 and 6). However, most two-part compound combinations showed additive or weakly antagonistic effects except the *t*-CA+PPA mixture against M. incognita, with the *t-*CA + indole combination showing the highest rate of antagonism against the two PPNs tested (Tables 5 and 6). These results indicate that there is an idiosyncratic interplay between the nematodes under study and the complex biochemistry of the tested SMs. Future studies should focus on the mechanisms of action of these *Photorhabdus* metabolites and their interactions on multiple nematode species. These interactions also raise interesting questions on the chemical ecology of *P. l. sonorensis* SMs in multitrophic interactions during the symbiotic life cycle of the bacterium. Additional investigations should also address the *in vivo* performance, stability, and toxicity of these SMs in greenhouse, and eventually, field studies.

### Conclusions.

Developing more effective, safe, and ecologically sustainable chemical control agents against nematode pests is a priority area for crop protection science. PPA, *t-*CA and indole show potent, concentration-dependent nematicidal, and/or nematistatic activities against two highly destructive PPNs; display promising selectivity in relation to model non-target species such as selected bacteria-feeding and entomopathogenic nematodes; and elicit negligible *in vitro* toxicity against human cells. Together with their accessibility via chemical synthesis, these properties warrant further investigations into these *Photorhabdus* SMs, formulated separately or as their synergistic mixtures, as lead compounds for nematicide development.

## MATERIALS AND METHODS

### Bacterial strains and cultivation conditions.

Photorhabdus luminescens *sonorensis* strain Caborca (46) was cultured on nutrient agar plates supplemented with 0.025% (wt/vol) bromothymol blue and 0.004% (wt/vol) 2,3,5-triphenyl tetrazolium chloride at 28°C in the dark (47, 48). A single blue-green, 40 h to 44 h old bacterial colony was inoculated into 15 mL Luria-Bertani (LB) medium in a 50-mL flask and cultivated with shaking at 200 rpm overnight at 28°C in the dark. One-milliliter aliquots of the resulting preculture were transferred into fresh LB media (100 mL LB in 500-mL Erlenmeyer flasks), and the cultures were incubated with shaking at 200 rpm for 96 h at 28°C in the dark.

### SM isolation and structure elucidation.

*P. l. sonorensis* cultures (10 L total volume) were acidified with 6 M HCl to pH 4.0 and extracted twice with equal volumes of ethyl acetate with shaking at 200 rpm for 30 min each at 28°C in the dark. The mixtures were centrifuged for 10 min at 5,000 rpm at 4°C, the organic phases were collected and combined, dried with 1% (vol/vol) anhydrous sodium sulfate, filtered through filter papers, and concentrated in a rotary evaporator at 37 to 40°C. The resulting thick oily, dark brown residues (crude extracts) were transferred to glass vials with lids and stored at 4°C in the dark.

For testing the nematicidal activity, the crude extracts were dissolved in dimethyl sulfoxide (DMSO) and diluted with distilled water to concentrations of 2,000 or 1,000 μg/mL (DMSO ≤ 1%; vol/vol). Nematicidal bioassays were implemented at each stage of the bioassay-guided isolation process and considered the J2 of the root-knot nematode, M. incognita, as the indicator organism. Crude extracts with demonstrated nematicidal activity were fractionated using open silica gel column chromatography (22 mm × 450 mm) with a stepwise gradient of a mixture of chloroform and methanol, starting at CHCl_3_:MeOH = 100:0, then 99:1, 98:2, 97:3, 95:5, 93:7, 90:10, 80:20, and 0:100, yielding 19 fractions (49). Each resulting fraction was evaluated for its nematicidal activity and monitored by thin-layer chromatography (TLC) and high-performance liquid chromatography (HPLC) following the procedure as described previously (23, 39).

Six fractions that showed nematicidal activity were further purified by preparative HPLC (Delta Prep 4000 system with a PDA 996 detector, Waters Corporation; equipped with a Kromasil 100 C_18_ reversed-phase column, 250 mm × 10 mm × 5 μm, Sigma-Aldrich). This led to the isolation of three metabolites with nematicidal activities (*t-*CA and PPA were present in more than one silica gel fractions). The isolated metabolites were identified as *trans*-cinnamic acid (*t-*CA; Fig. 1 and Table S1), (4*E*)-5-phenylpent-4-enoic acid (PPA; Fig. 1 and Table S2), and indole (Fig. 1 and Table S3). The chemical structures and purities of the isolated metabolites were determined by nuclear magnetic resonance (NMR) spectroscopy (one dimensional ^1H-NMR^ and ^13^C-NMR spectra), and liquid chromatography-mass spectrometry (LC-MS) to determine the molecular weight and the ion fragmentation patterns. Structure assignments were further confirmed by comparing the isolated SMs with chemically synthesized authentic standards from commercial sources (“synthetic SMs”: *t-*CA and indole from Alfa Aesar, and PPA from Enamine). In addition, the nematicidal activities of the isolated SMs and the commercially available synthetic SMs were also compared and validated to be equipotent in causing mortality.

### Nematode rearing.

Only soil inhabiting (free-living or infective) stages of target and non-target nematode species were considered in the present study. All nematode cultures (except *T. semipenetrans*) were maintained in the Stock laboratory (University of Arizona). M. incognita was reared *in planta* using susceptible tomato plants (cv. Roma VF; seedlings with five or six true leaves; courtesy of Dr. J. Brown, University of Arizona). Eggs of M. incognita were extracted from infected tomato plant roots, and J2 were collected after hatching, following the procedures of Atamian et al. (50). *T. semipenetrans* was isolated from infested lemon orchards at the Yuma Agricultural Center, University of Arizona (Yuma, AZ, USA). Eggs of *T. semipenetrans* were extracted from infected lemon tree roots using the procedures of El-Borai et al. (51), and J2 were collected using the same procedure as that described for M. incognita.

The non-target species, C. elegans wild-type strain N2 Bristol (courtesy of Dr. G.L. Sutphin, University of Arizona) and *Rhabditis blumi* DF5010 (*Caenorhabditis* Genetics Center, University of MN, USA) were maintained *in vitro* in nematode growth medium (NGM) agar plates seeded with Escherichia coli (Migula) Castellani and Chalmers, strain OP-50, as described previously (52). Mixed juveniles and hermaphrodites (5 to 6 days old) were collected from the culture plates prior to the bioassays. Infective third-stage juveniles (IJs) of three non-target entomopathogenic nematode species (*H. sonorensis* Caborca strain, *H. bacteriophora* TT01 strain, and Steinernema carpocapsae All strain) were also maintained by rearing them *in vivo* using wax moth larvae (Galleria mellonella) as the host, according to procedures described previously (53), and collected for bioassays within 2 to 4 days of their emergence from nematode-infected cadavers. Nematode population density (number of nematodes per ml) was standardized prior to setting up well-plate bioassays, by adjusting each nematode suspension to a concentration of 100 nematodes in 20 μL of distilled water.

### Nematicidal assays.

Twelve-well tissue culture plates were used as the experimental arena. For each of the tested nematode species, an inoculum of 100 nematodes, suspended in 20 μL distilled water, was added to each well. For *t-*CA (Alfa Aesar), 14 incremental concentrations were considered, ranging from 10 to 500 μg/mL in distilled water, with a final volume of 1 mL/well (DMSO ≤ 1%; vol/vol). For PPA (Enamine) and indole (Alfa Aesar), 13 incremental concentrations were tested ranging from 10 to 400 μg/mL in 1 mL of distilled water (DMSO ≤ 1%; vol/vol). Due to the relative insolubility of PPA and indole in water, higher concentrations 400 μg/mL than could not be considered. Distilled water with 1% DMSO was used as the negative control. To measure the combined nematicidal effects of the three SMs, equal concentrations of two or three SMs were mixed in 1:1 or 1:1:1 mixture. For example, a 20 μg/mL solution of a two-SM mixture contained 20 μg from metabolite A and 20 μg metabolite B, dissolved in 1 mL distilled water. Individual or combined SMs from 10 μg/mL to 200 μg/mL (in 20 μg/ml increments) were prepared in distilled water (DMSO ≤ 1%; vol/vol) and used for the bioassays.

The well plates were covered with their lids and incubated at 25°C in the dark. For the targeted nematode species, nematicidal activity was recorded at 24 h, 48 h, and 72 h after initial exposure to the individual SMs. For the non-target species, data were only recorded at 24 h after initial exposure to the individual SMs. For SM mixtures, mortality was recorded 24 h after initial exposure of the targeted nematode species. Nematodes were probed with a needle to assess if they were alive, dead, or paralyzed. Typically, dead nematodes were straight and had a clear appearance due to cell necrosis, while paralyzed nematodes had a shrunken, wavy, curved, or rounded appearance. Those nematodes that resumed activity upon probing were considered to suffer from temporary paralysis (impaired motility). Paralyzed nematodes were rinsed at least three times in distilled water and held for an additional 24 h to check for their recovery from temporarily paralysis. To ascertain the LC_50_ of indole, dilutions from 260 μg/mL to 400 μg/mL (in 20 μg/mL increments) were used (DMSO ≤ 1%; vol/vol). Experiments were repeated at least three times per treatment for each concentration tested.

### Cytotoxicity assays.

The non-cancerous HFF cells (human foreskin cells) and three human cancer cell lines, NCI-H460 (non-small cell lung cancer), SF-268 (central nervous system glioma), and MCF-7 (breast cancer) were used to evaluate the *in vitro* cytotoxicity of the isolated *Photorhabdus* SMs. The MTT (3-(4,5-dimethylthiazol-2-yl)-2,5-diphenyltetrazolium bromide) colorimetric method (54) was used with doxorubicin as the positive control, and DMSO as the negative control. *Photorhabdus* SMs were tested at 50, 100, and 200 μM concentrations, and the assays were repeated three times.

### Data analysis.

For the toxicity data visualization, the relationship between percent mortality or temporary paralysis and metabolite concentration were described by a sigmoidal curve, which was fitted by the logistical function using the statistical software package SigmaPlot for Windows, version 14.0 (Systat Software Inc.). The inflection point of the sigmoidal curve corresponds to the concentration that kills or immobilizes 50% of the test population (lethal concentration, LC_50_; or effective concentration of temporary paralysis, EC_50_). Concentration-response data of single metabolites or SM mixtures were pooled for each concentration and subjected to probit regression analysis using the Polo Plus program (LeOra Software). The LC or EC values, along with the corresponding 95% confidence intervals, were estimated for each single SM or SM mixture at each exposure time, depending on the experimental setting.

The selectivity index of a given SM was calculated by dividing the averages of the LC_50_ or EC_50_ values obtained against each of the selected non-target nematodes by the LC_50_ or the EC_50_ values observed for each of the targeted PPN tested.

For the analysis of SM combination effects, the statistical method described by DeLorenzo and Serrano (43) was considered:

$$\text{S }=\;\left({\text{A}}_{\text{m}}/{\text{A}}_{\text{i}}\right)+\left({\text{B}}_{\text{m}}/{\text{B}}_{\text{i}}\right)$$

Where: S = sum of biological activity; A_m_ = LC_50_ for compound A in the mixture; A_i_ = LC_50_ for compound A measured alone (individual effect); B_m_ = LC_50_ for compound B in the mixture; B_i_ = LC_50_ for the individual effect of compound B. S values were used to calculate an additive index (AI). If S ≤ 1.0, then AI = (1/S) − 1.0. If S ≥ 1.0, then AI = S(–l) + 1.0. An AI value with confidence intervals in the negative range indicates antagonistic toxicity; an AI value with confidence intervals greater than zero indicates synergistic toxicity; and an AI value with confidence intervals overlapping zero indicates additive toxicity.

Statistically significant differences in LC or EC values among SMs at a given exposure time, within or among nematode species, were first interpreted based on non-overlapping 95% confidence intervals. In addition, averages of LC or EC values of each replicate from each single SM and mixtures were used to perform unpaired two-samples Wilcoxon tests via the NPAR1WAY procedure of SAS to compare and assess selective effects of each SM between the target and non-target nematodes species, within the target nematodes, or within the non-target nematode species. For the nematicidal or nematistatic time course studies with single SMs, data from the 24 h, 48 h, and 72 h exposures were used for a linear regression analysis with the REG procedure and unpaired two-samples Wilcoxon tests with the NPR1WAY procedure in SAS. To further evaluate the interaction effects among the seven different SM applications (single compounds and two-part or three-part compound mixtures), the LC_50_ values were compared using one-way ANOVA with the GLM procedure of SAS, followed by an orthogonal contrasts test. All analyses were performed using SAS for Windows version 9.4 (SAS Institute Inc.).

### Data availability.

The data sets generated during and/or analyzed during the current study are available from the corresponding author on reasonable request.
